# Anti-inflammatory and arthritic activity of zaltoprofen compared to piroxicam in murine models

**DOI:** 10.6026/97320630018752

**Published:** 2022-09-30

**Authors:** Balakrishnan Sadasivam, Ratinder Jhaj, Saman Kumar, Saman Pathan, Santenna Chenchula

**Affiliations:** 1 Department of Pharmacology, All India Institute of Medical Sciences, Saket Nagar, Bhopal - 462020, Madhya Pradesh, India

**Keywords:** Acute Inflammation, Chronic Inflammation, Arthritis, Carrageenan, Zaltoprofen; Piroxicam

## Abstract

Zaltoprofen, a unique propionic acid group of NSAIDs, works by blocking the enhancing effects of bradykinin along with the COX-2 enzyme. Therefore, it is of interest to evaluate the acute and chronic anti-inflammatory (arthritic) potential of zaltoprofen versus piroxicam in Murine models. A total of 48 Wister rats (200-250 g) of either sex (24 in each model) were used in the present study. The anti-inflammatory and arthritic potential of zaltoprofen was evaluated and compared by Carrageenan-induced acute inflammation and formalin-induced chronic inflammation. There was a significant inhibition of paw volume (P<0.001) on different time scales with two different doses of the test compound (Zaltoprofen 10 & 20 mg/kg) in the acute inflammation model compared to the negative control (NaCl 10 ml/kg). However, in the chronic inflammation model, zaltoprofen 10 mg/kg and 20 mg/kg doses of the test compound showed a significant reduction in chronic inflammation, comparable to the negative control (NaCl 10 ml/kg), although the potency was lower than the positive control (piroxicam 10 mg/kg) (P 0.05). Thus, zaltoprofen shows significant anti-inflammatory and arthritic effects in both acute and chronic models by inhibiting various inflammatory mediators.

## Background:

Inflammation is an essential biochemical protective response of vascularized tissues that delivers leukocytes and molecules of host defence from the circulation to the sites of infection and cell damage to eliminate the offending agents [1]. It serves to clear the host of both the initial cause of cell injuries like microorganisms and toxins and the consequences of such injury, which may be necrosis of cells and tissues. Various mediators of defence include phagocytic leukocytes, antibodies, and complement proteins. During the inflammation, circulating inflammatory cells and proteins reach the injured tissues and activate the recruited and resident cells, as well as the soluble molecules, which could help to get rid of harmful or unwanted substances. Inflammation is essential for survival to overcome infections and heal wounds. Acute inflammation develops within some minutes of tissue injury or infection, having characteristics of fluid and plasma proteins exudation, leading to oedema, and the migration of leukocytes, primarily neutrophils, and is very short in duration. However, if the offending stimulus is removed, the reaction subsides, and residual injury is repaired. Rheumatoid arthritis (RA) is a type of chronic inflammation, which is longer in duration and is associated with more tissue destruction and scarring [1]. Both Steroidal and nonsteroidal anti-inflammatory drugs (NSAIDs) are the most commonly used drugs for the prevention and treatment of inflammation. However, in long-term use, NSAIDs are associated with gastrointestinal, hepatic, renal and cardiac side effects, etc., [2]. Hence, it is very essential to find drugs with lower side effects for the treatment of inflammatory diseases. Zaltoprofen is a unique NSAID from the propionic acid group with a tri-cyclic formula. Its chemical name is 2-(6-oxo-5H-benzo[b][1] benzothiepin-3-yl) propanoic acid [3]. It has a potent analgesic and antipyretic activity [4-6]. It acts by blocking the, augmenting effect of bradykinin a potent endogenous algesic or sensitizing substance, and acts through Bradykinin-1(inducible) and Bradykinin-2(constitutive) receptors. Zaltoprofen acts by inhibiting Gq protein-coupled B2 receptor-mediated action on α-amino-3-hydroxy-5-methyl-4-isoxazole propionic acid receptor (AMPA) currents, through the inhibition of protein kinase-C activation in the primary sensory neurons [7]. Additionally, several different signal transduction pathways, including phospholipase C (PLC), phospholipase A2, lipooxygenase (LOX), and cyclooxygenase (COX) enzymes in dorsal root ganglion (DRG) cells [7]. Zaltoprofen also exhibits an anti-inflammatory effect by inhibiting the cyclooxygenase-2(COX2) enzyme selectively and inhibits prostaglandin-E2 production at inflammatory sites [6-8]. Previous studies have shown that zaltoprofen has an anti-inflammatory action at ED50, 1-5 mg/kg, per oral [4]. Hence we have planned to study the dose range above 5 mg/kg body weight. In addition, till now no studies comparing the anti-inflammatory and arthritic activity of zaltoprofen over the most common prescribing long-acting NSAID piroxicam. Therefore, it is of interest to evaluate and compare the anti-inflammatory and arthritic activity of Zaltoprofen efficacy over piroxicam in the Murine models of acute and chronic inflammation or arthritis.

## Materials and Methods:

### Experimental Animals:

A total of 48 male Wistar albino rats (200-250 g) were used for the experimentations which were obtained from the Central Animal House of the institution, All India Institute of Medical Sciences (AIIMS) Bhopal, India. All the animals were adapted to the new environment minimum of two weeks before initiating the experiments using six animals in each group. The animal house temperature was maintained at 25°C ± 2°C with 12 hours of light and 12 hours day cycle and they were allowed to have free access to a standard pellet diet and water. The present experimental study was carried out as per the Committee for the purpose of control and supervision of experiments on animals(CPCSEA) guidelines after the approval by the Institutional Animal Ethics Committee (IAEC), AIIMS Bhopal, India (AIIMS/BHOPAL/IAEC/2017/03 Date: 10th August 2017).

### Chemicals and drugs:

Chemicals Carrageenan and Formalin were obtained from Himedia Laboratories Pvt. Ltd (Mumbai, India). The Test compound Zaltoprofen was obtained from JB Chemicals and Pharmaceuticals Ltd (Mumbai, India) and the standard agent Piroxicam from Medipol Pharmaceutical India Pvt. Ltd. (New Delhi, India).

### Experimental Dosing:

All the 48 experimental rats were randomly divided into four different groups (n=6 in each group) in each model. Group I (Negative Control) were treated orally with normal saline (NS) 10 ml/kg body weight. Group II (Positive Control) were treated orally with piroxicam 10 mg/kg body weight (PRC10). Group III (Test-I) were treated orally with Zaltoprofen 10 mg/kg body weight (ZLT10). Group IV (Test-II) were treated orally with Zaltoprofen 20 mg/kg body weight (ZLT20).

### Experimental induction of acute and chronic inflammation:

Carrageenan-Induced acute inflammation: Carrageenan-induced paw oedema is the unique model of acute inflammation having large reproducibility. Inflammation was induced in rats according to previous standard research protocols [9] as shown in Figure 1(see PDF). Six animals of either sex in each group were used in the total four groups. Rats were administered orally 0.9% NaCl (10ml/kg), zaltoprofen (10 mg/kg and 20 mg/kg), and piroxicam (10 mg/kg) dissolved in 0.9% normal saline, 1 hour before carrageenan administration. After that 0.1 ml of 1% carrageenan dissolved in normal saline was injected subcutaneously into the right hind paw for the induction of acute inflammation. The average volume of the left hind paw of each mouse was measured before injecting carrageenan, and at 30 minutes, 1 hour, 2 hours, and 3 hours after injection of carrageenan, using a digital plethysmometer (ORCHID Scientific & Innovative India, Pvt. Ltd. India) [9]. By using the following formula we have calculated the percentage of inhibition in comparison with the control group.

% Inhibition = (Vd -Vb /Vb) x 100. Vd is the paw volume at different times; Vb is the paw volume before inflammation was induced

### Formalin-induced arthritis:

In the formalin-induced chronic inflammation model, paw oedema was induced by subaponeurotic administration of 0.1 ml of 2% formaldehyde at 30 minutes after the oral administration of standard/test/vehicle compounds on day 1 and day 3 respectively. Experimental animals were administered daily, with Tests I & II (Zaltoprofen (10mg/kg and 20 mg/kg) and Standard (Piroxicam 10 mg/kg) agents orally for 10 days. The daily changes in paw oedema or linear cross-section (LCS) of each rat, below the ankle joint, were measured daily with a digital vernier calliper [1].The difference in the paw volume on day 1 and day 10 was considered inflammatory oedema. Volume changes in Test I, Test II and standard groups were compared with that of control and percentage inhibition was calculated. The percentage inhibition of the inflammation was determined by the formula: % Inhibition = 1- (Vt/Vc) x 100. Vc is the oedema volume in control and Vt is the oedema volume in the group treated with the tested Zaltoprofen or Piroxicam.

### Statistical procedure:

Data were analyzed using SPSS version 22.0. Results were expressed as mean ± SD. The percentage inhibition of the inflammation was calculated for each model. Comparison between all the four different groups was done by using one-way ANOVA followed by Bonferroni's test to compare the difference between groups at different time intervals. A P-value less than 0.005 was considered statistically significant when compared with control (*p <0.005).

## Results and Discussion:

### Acute anti-inflammatory activity:

The mean of the basal paw oedema volume was comparable in all the groups. After 60 and 120 minutes, the mean decrease in paw volume in all three groups (ZLT10mg/kg, ZLT 20 mg/kg & PRC10mg/kg) was statistically significant when compared to the negative control group (P < 0.001). For the negative control group, the injection of carrageenan caused localized oedema, after 30 min. The swelling increased progressively from 30 min onwards. A maximum volume of the paw was observed in animals of negative control at 120 min (2.31±0.348) (0.289 ± 0.008 ml) after injecting carrageenan. Test-I (zaltoprofen 10mg/kg) and Test-II (zaltoprofen 20 mg/kg) caused significant decrease in the paw volume (***P= <0.001) compared to control (1.43±0.068 and 1.36 ± 0.128) with 38.24% and 40% inhibition of inflammation respectively (Table 1 - see PDF). The standard agent (piroxicam20mg/kg) significantly reduced (***P < 0.001) the paw oedema (1.28±0.088) with 44.52% inhibition of inflammation. Percentage inhibition of acute inflammation was greater in the standard drug piroxicam group (Positive Control) compared to Test groups I & II (ZLT 10mg/kg and ZLT 20mg/kg), at all-time intervals. Similarly, the percentage inhibition in the Test-II (ZLT 20mg/kg) group was higher than that of Test-I (zaltoprofen 10mg/kg) at all-time intervals (Figure 2 - see PDF).

### Chronic anti-inflammatory (arthritic) activity:

Administration of formalin caused an increase in paw thickness in all groups. In the case of negative control, the highest increase in paw thickness was observed on day 3 and slowly reduced, but not restored to its original paw thickness which was on day 1. Table 1(see PDF) depicts the mean± SD difference On the Linear Cross-Section (LCS) below the Ankle Joint, for each group. The lower difference is increase in paw volume (LCS), the higher the anti-inflammatory action. The mean difference in LCS in all the three drug-treated groups was significantly lower when compared to Negative Control (P < 0.001), though the mean difference in LCS was higher in Test I and II (zaltoprofen 10 and 20 mg/kg) than that in the piroxicam group (Positive Control). The lowest difference in LCS was found in the Positive Control (piroxicam 10mg/kg) group. Formalin-induced paw oedema was reduced significantly (p<0.001) with the administration of Test I and Test II (zaltoprofen 10 mg and 20mg), which was found to be a time-dependent and the highest reduction in paw thickness was observed on day 7 and (zaltoprofen10mg/kg) Day 5 (Zaltoprofen 20mg/kg) respectively (Table 2 - see PDF). Percentage inhibition of chronic inflammation was higher in the piroxicam group (35.60%) when compared to Test-I (18.90%) and Test-II (27.76%) at all-time intervals (Figure 3 - see PDF). Similarly, the percentage inhibition in Test-II (zaltoprofen20mg/kg) group was higher than that of Test -I (zaltoprofen 10mg/kg) at all-time intervals.

## Discussion:

The present experimental study has compared the acute and chronic anti-inflammatory or arthritic potential of Zaltoprofen, using well-established experimental murine models. The Test drug Zaltoprofen was investigated and compared over piroxicam by using two different doses of the Test compound (ZLT 10 and 20 mg/kg vs. PRC10 mg/kg) in carrageenan-induced acute inflammation and formalin-induced chronic inflammation or arthritis models, and the results were depicted fig. Carrageenan-induced acute inflammation is a standard experimental model for acute inflammation. A major advantage of carrageenan is, that single oral doses of drugs at non-toxic levels are effective; and it is similar to the early exudative stage of inflammation in humans and the model is linked with the activation of various inflammatory autacoids like histamine, serotonin, bradykinin, prostaglandins and leukotrienes. After injecting the carrageenan into the hind paw it induces progressive oedema that reaches its maximum within 3 hours [9]. Both test doses of zaltoprofen showed significant anti-inflammatory activity in comparison to the negative control (NaCl 10ml/kg). However, when compared to a positive control (Piroxicam 10mg/kg) anti-inflammatory activity of test doses (TEST I & II) of zaltoprofen were less than standard piroxicam. Inhibition of carrageenan-induced oedema by zaltoprofen can be attributed to its ability to inhibit the various mediators of inflammation like kinins and prostaglandins. Formalin-induced chronic inflammation is a model for arthritis and is considered the most suitable experimental animal model which closely resembles arthritis in human beings. It is associated with the proliferative phase of chronic inflammation in human beings and is biphasic, an early neurogenic phage is mediated by substance-P and bradykinin followed by a later tissue-mediated response where, histamine, 5-hydroxytryptamine (5-HT), prostaglandins (PGs) and bradykinin are known to be involved [10]. Zaltoprofen has shown a significant inhibition in paw volume in the formalin-induced chronic inflammation or arthritis model. Test-I (ZLT 10mg/kg) inhibited 19% of paw oedema, Test-II (ZLT 20 mg/kg) inhibited 28% of paw oedema, and standard treatment piroxicam decreased a total of 36% of paw oedema respectively. Both test doses showed significant antiarthritic activity, although the effect was lower than the positive control (PRC 10 mg/kg). As this model is more appropriate and very commonly used to evaluate anti-inflammatory agents with probable anti-proliferative activity, zaltoprofen's inhibitory effect on formalin-induced chronic inflammation indicates its significant anti-proliferative and arthritic activity [11].

An In vivo study by Tang et al. has shown that Zaltoprofen inhibits bradykinin-induced 12-lipoxygenase (LOX) activation in vitro and the slow bradykinin-induced onset of substance-P release from dorsal root ganglion (DRG) neurons [12]. A study by Matsumoto et al. concluded that Zaltoprofen has inhibitory action specifically on bradykinin-induced nociception in nociceptor endings through B2-type bradykinin receptors [13].Findings of an experimental study by Kameyama et al. has been shown that zaltoprofen inhibits bradykinin-induced nociceptive responses more significantly, with a better safety margin, having less potential to induce gastric ulcers than indomethacin [6]. Another study by Santenna et al. concluded that zaltoprofen analgesic efficacy was non-inferior to a standard NSAID piroxicam in experimental murine models of acute pain [5]. A study by Muratani et al. has shown thatzaltoprofen produces the preemptive analgesic effect peripherally by blocking the B2 pathway [14]. A multicentric, double-blind, double-dummy, randomized, parallel-group comparative study by Pareek et al. concluded that clinically zaltoprofen is non-inferior to diclofenac [15]. A spate of previous studies has shown that zaltoprofen might be a potential agent to act against malignant phenotypes in chondrosarcomas via activation of PPAR γ and inhibition of MMP 2 activity [15-16]. A randomized placebo-controlled, double-blind, phase II trial by Akihico et al. found that Zaltoprofen can be a potential newer therapeutic agent, to stabilize disease progression for patients with diffuse tenosynovial giant cell tumour (D-TGCT) or unresectable L-TGCT [17]. A study by Okamoto et al. reported that Zaltoprofen improves the loss of body weight in both the Con A-induced mouse and carbon tetrachloride-induced rat sickness behaviour models, like other commonly used NSAIDs Aspirin and Indomethacin [1]. A non-comparative multicentre study by Hatori et al., among rheumatoid arthritis patients, has shown that, in the long-term treatment of rheumatoid arthritis, zaltoprofen is a well-tolerated and safe non-steroidal anti-inflammatory agent [18]. Zaltoprofen is predominantly metabolized by CYP2C9 and UGT2B7 and is unlikely to cause significant drug interactions in vivo when co-administered with CYP substrates at clinically effective doses [19]. Though the study findings have shown a significant anti-inflammatory activity of zaltoprofen, our study suffered from some limitations: we have not done any Histopathological estimation of inflammatory biomarkers in relation to acute and chronic inflammation.

## Conclusions:

Data shows that zaltoprofen in a dose-dependent manner is an effective agent in attenuating carrageen and formalin-induced acute and chronic inflammation and arthritis cascade in experimental murine models of inflammation or arthritis. Thus, it could be considered a potential therapeutic agent for acute and chronic inflammatory clinical conditions in humans, particularly those associated with arthritis. Further evaluations are necessary on the effect of zaltoprofen on histopathological and biomarker changes of acute and chronic inflammation.

## Authorship contribution statement:

Concept and idea was from Santenna Chenchula and Balakrishnan Sadasivam. Santenna Chenchula, Balakrishnan and Sunil Kumar conducted the literature search and animal experiments. Santenna, Ratinderjhaj and Saman Pathan did statistical calculations and drafted the manuscript. Ratinderjhaj and Balakrishnan revised the final manuscript. All authors reviewed and approved the final version of the manuscript.
